# Supplementary material for: Exposure to environmental pollutants selects for xenobiotic-degrading functions in the human gut microbiome
Source: Nat Commun. 2024 May 27;15:4482. doi: 10.1038/s41467-024-48739-7 (PMC11130323; doi:10.1038/s41467-024-48739-7)
Supplement: Supplementary file 1 — Supplementary Information [file 41467_2024_48739_MOESM1_ESM.pdf]

SUPPLEMENTARY INFORMATION

Exposure to environmental pollutants selects for xenobiotic-degrading functions in the human gut microbiome

Francesca De Filippis, Vincenzo Valentino, Giuseppina Sequino, Giorgia Borriello, Marita Georgia Riccardi, Biancamaria Pierri, Pellegrino Cerino, Antonio Pizzolante, Edoardo Pasolli, Mauro Esposito, Antonio Limone, Danilo Ercolini

SUPPLEMENTARY FIGURES

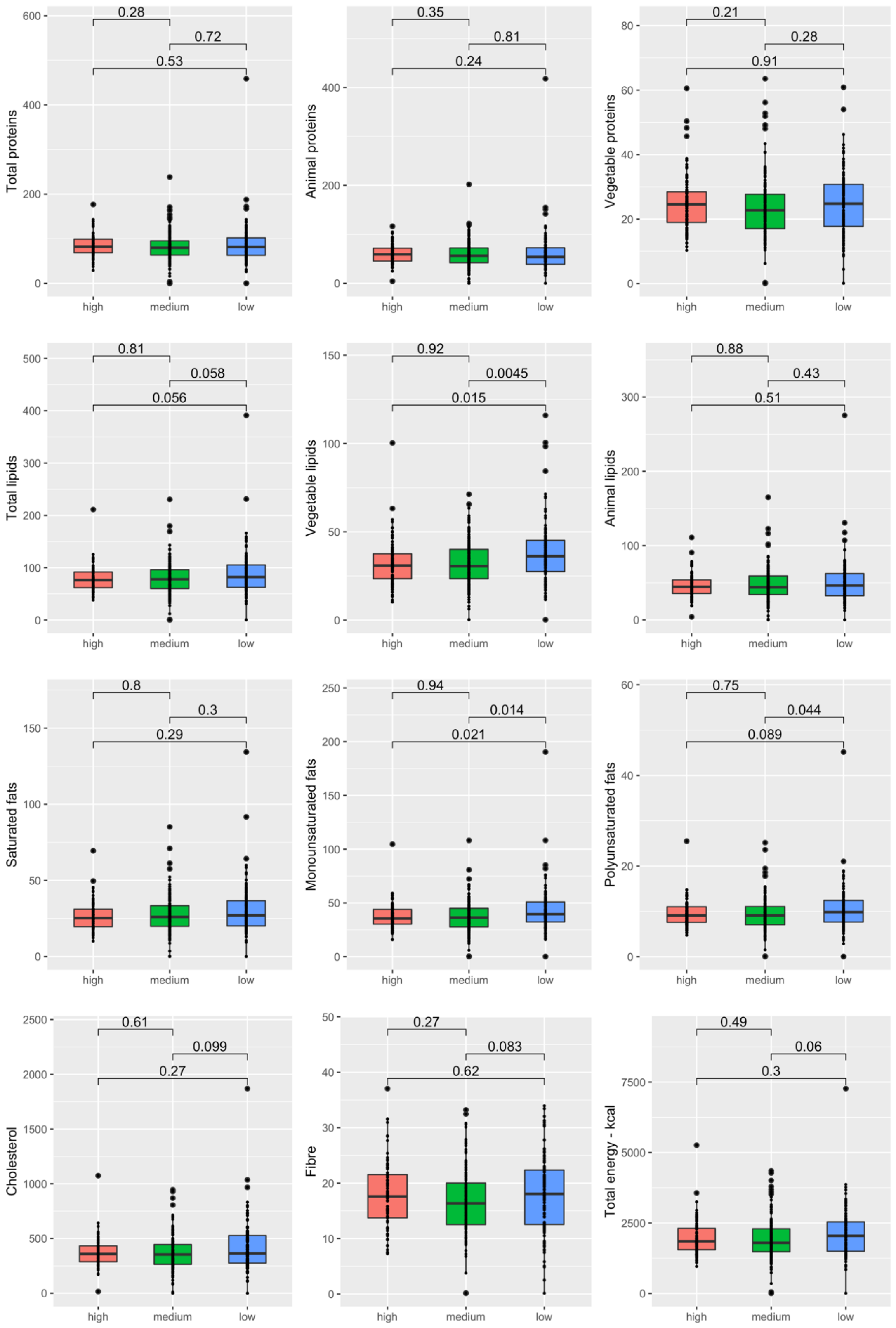

Figure S1. Box plots showing the abundance in the habitual diet of the main macronutrients, in subjects from areas at HIGH, MEDIUM and LOW environmental pressure, as defined by MIEP

index. The significance was tested by applying pairwise Wilcoxon test with p-value correction using the False Discovery Rate approach. Data are obtained from n= 82, 161 and 116 biologically independent samples from HIGH, MEDIUM and LOW environmental pollution groups, respectively.

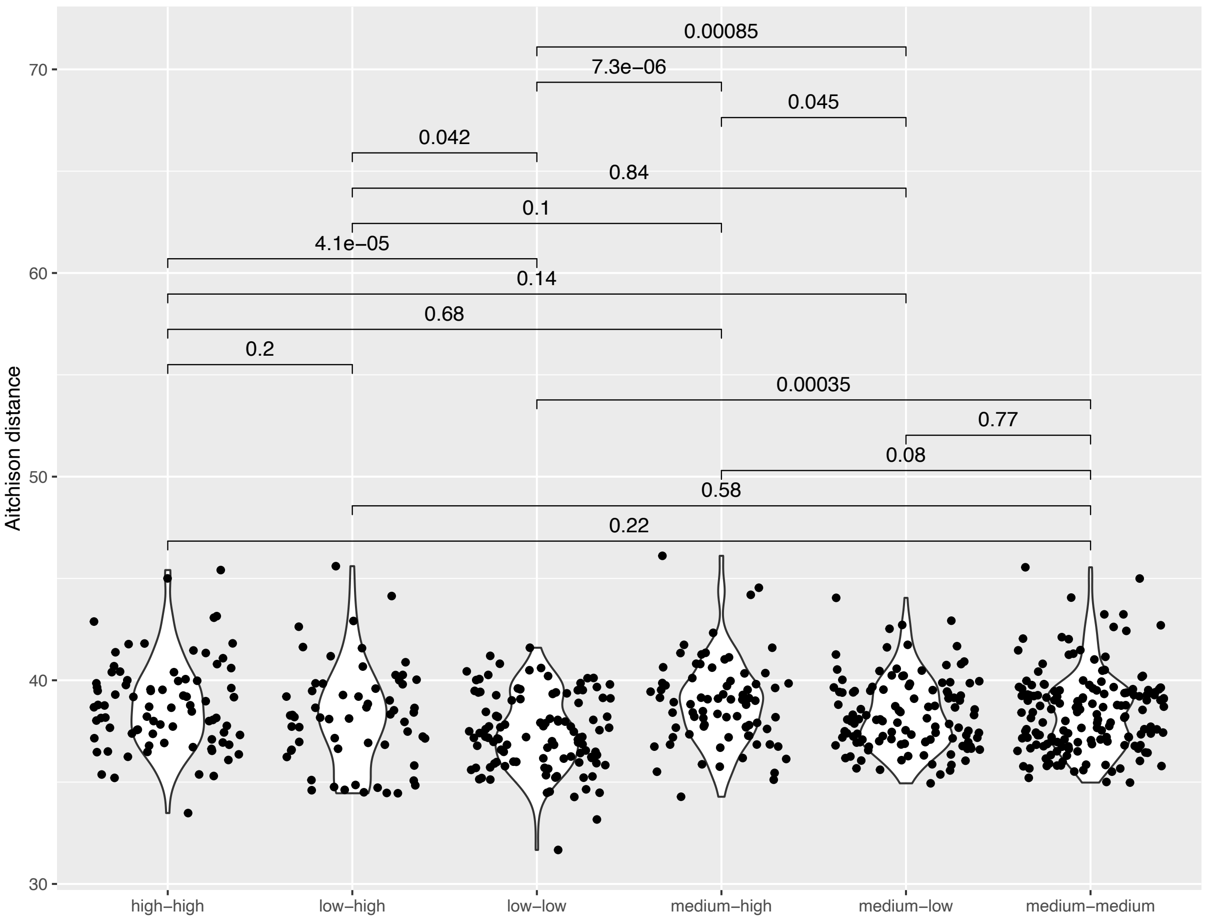

Figure S2. Violin plots showing Jaccard's distance based on gut microbiome composition between groups in subjects from areas at HIGH, MEDIUM and LOW environmental pressure, as defined by MIEP index. Data are obtained from n= 82, 161 and 116 biologically independent samples from HIGH, MEDIUM and LOW environmental pollution groups, respectively.

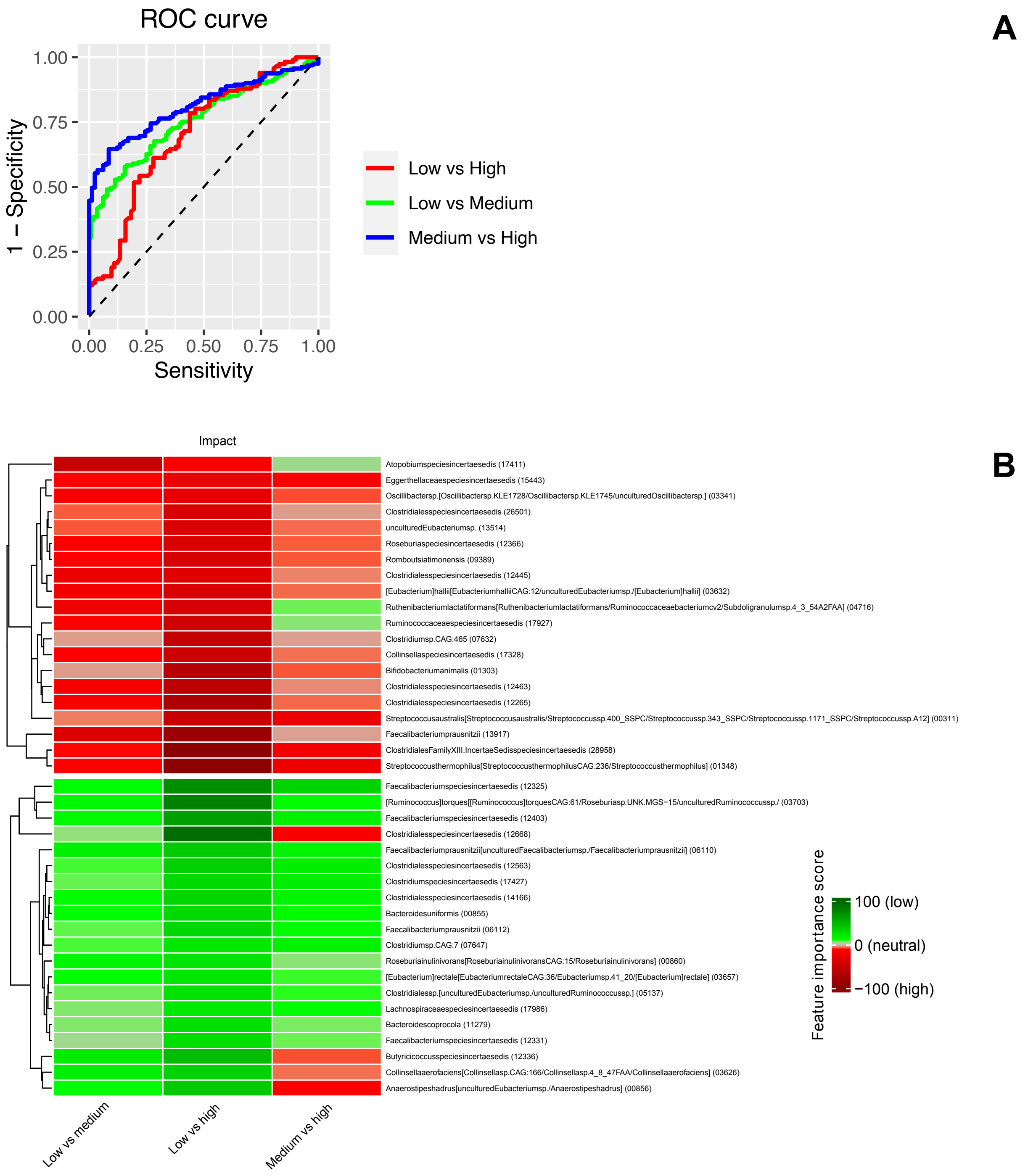

Figure S3. Random Forest analysis on microbiome taxonomic composition. (A) Receiver operating characteristic (ROC) curves for the three tested scenarios. The results were obtained by RF classification, and averaged over folds and repetitions. (B) Feature importance score as computed by RF classification across the three tested scenarios. We report the top-20 features (taxa) associated with a higher (in red) or lower (in green) impact for each couple of impact area.

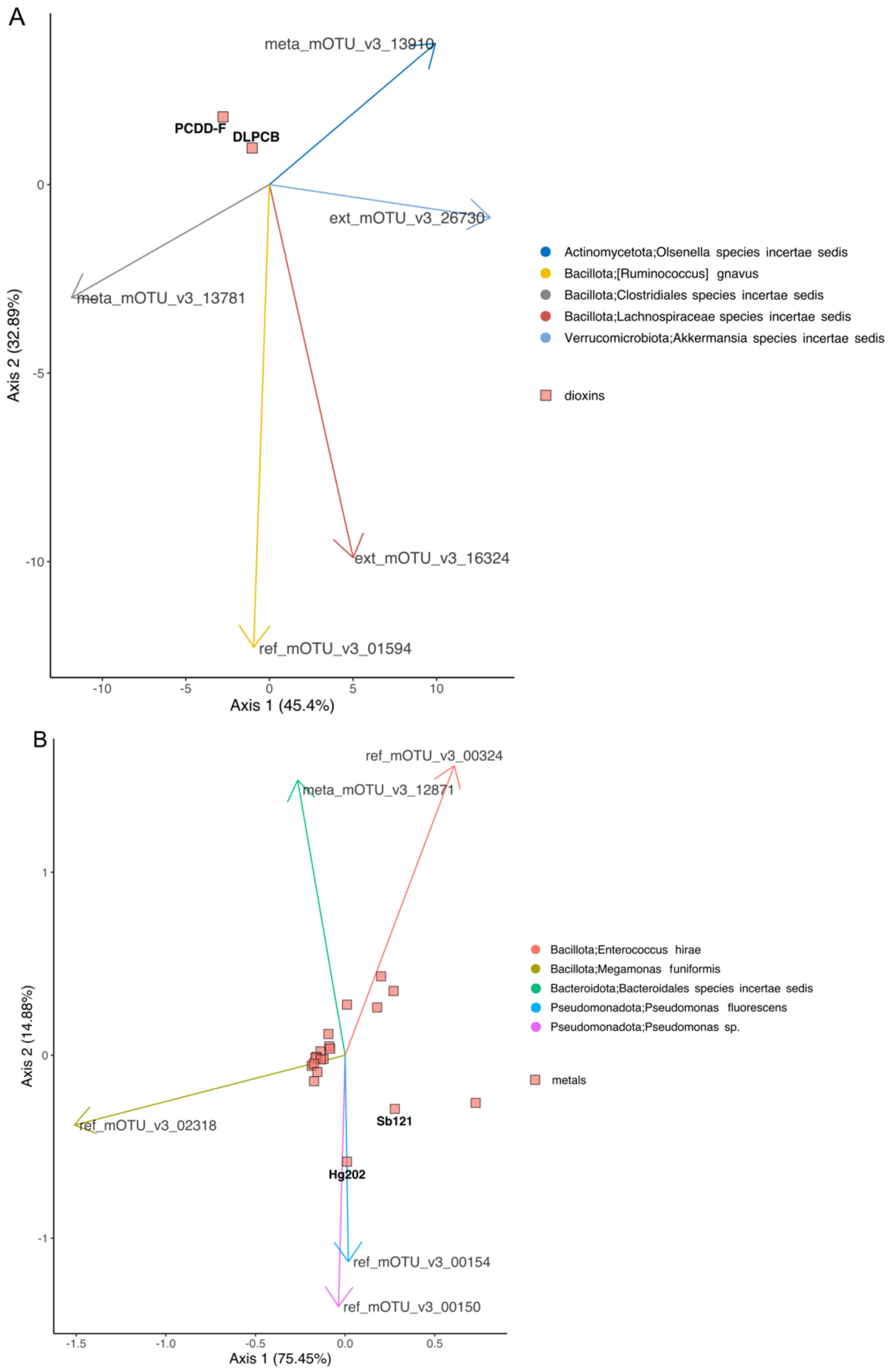

Figure S4. Biplots of interactions between microbes and dioxins (A) or metals (B), as estimated by *mmvec*. Arrows represent mOTUs and are color-coded according to the taxonomy, whereas red squares represent dioxins (A) or metals (B). Arrows and squares pointing in the same direction are indicative of their co-occurrence.

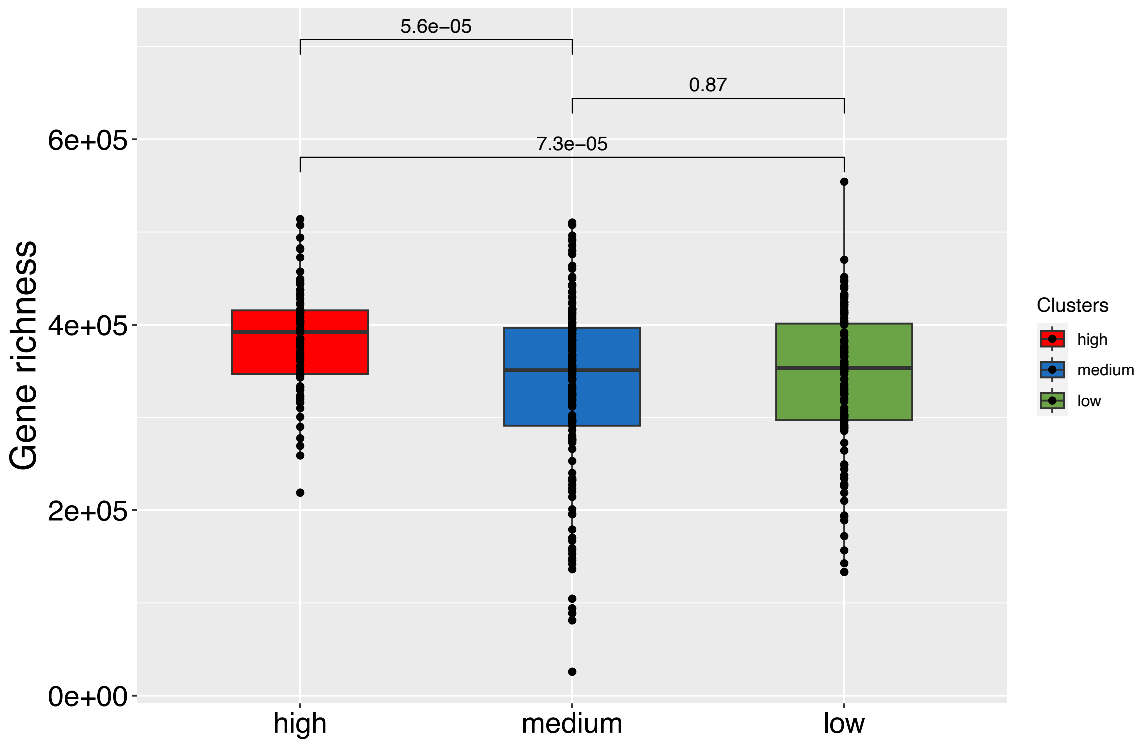

Figure S5. Environmental pollution increases gut microbiome functional diversity.

Box plots showing gene richness in subjects from areas at HIGH, MEDIUM and LOW environmental pressure, as defined by MIEP index. Boxes represent the interquartile range (IQR) between the first and third quartiles, and the line inside represents the median (2nd quartile). Whiskers denote the lowest and the highest values within 1.5 x IQR from the first and third quartiles, respectively. The significance was tested by applying pairwise Wilcoxon test with p-value correction using the False Discovery Rate approach. Data are obtained from n= 82, 161 and 116 biologically independent samples from HIGH, MEDIUM and LOW environmental pollution groups, respectively.

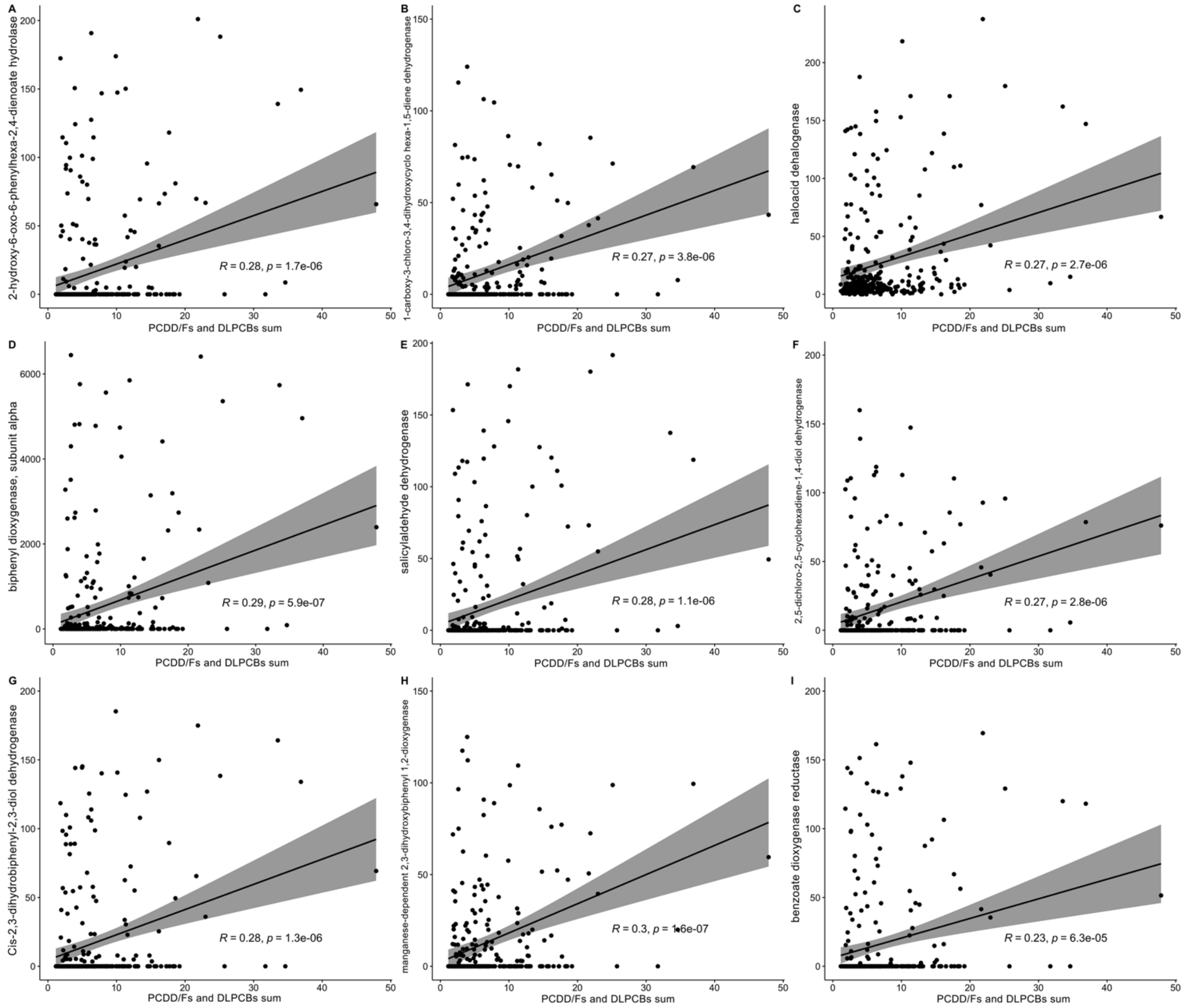

Figure S6. Spearman's correlations between PCDD/Fs + DLPCB (Medium Bound; pg WHO-TEQ/g lipids) concentration and the abundance (RPKM) of several genes related to dioxin degradation pathways.

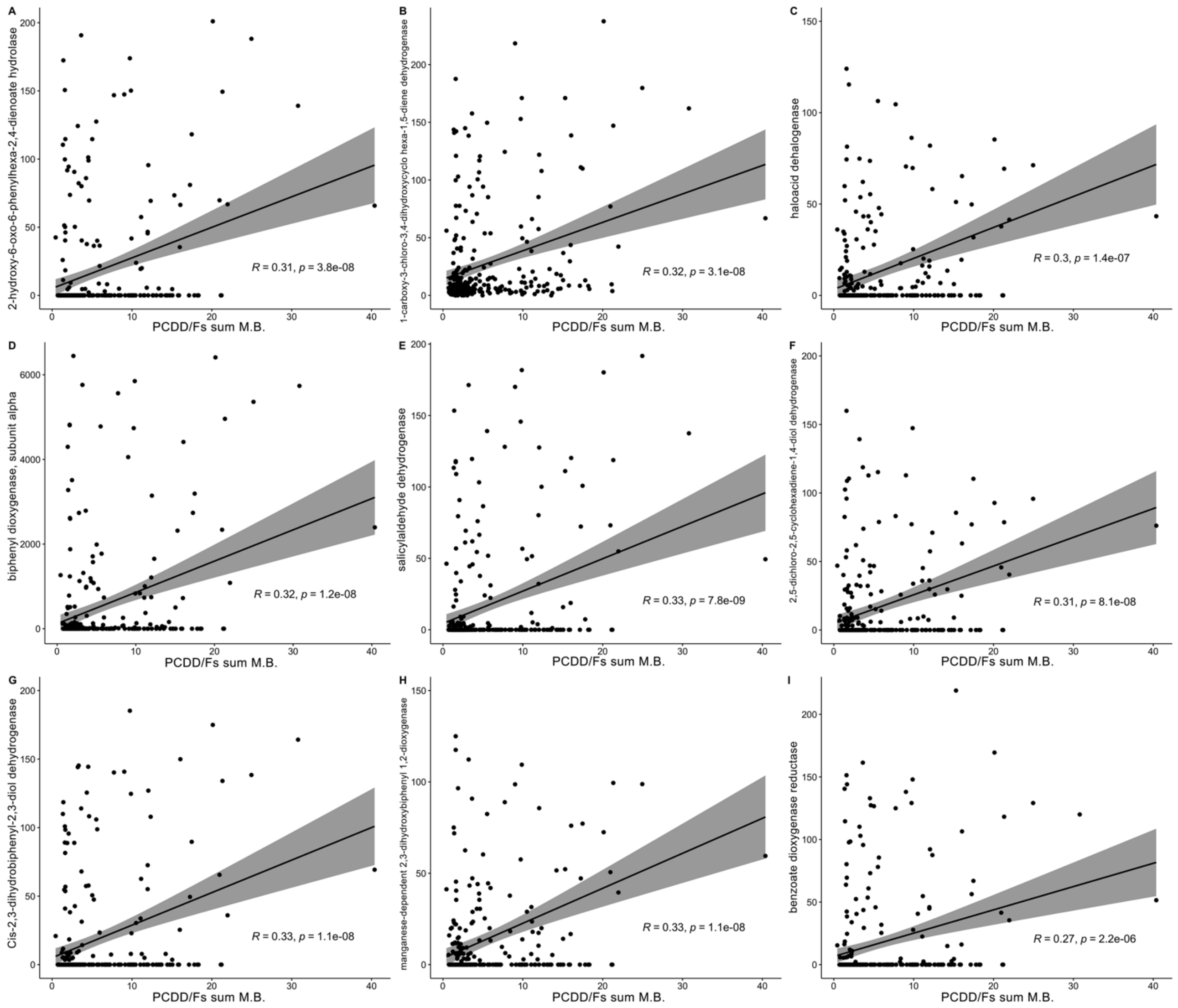

Figure S7. Spearman's correlations between PCDD/Fs (Medium Bound; pg WHO-TEQ/g lipids) concentration and the abundance (RPKM) of several genes related to dioxin degradation pathways.

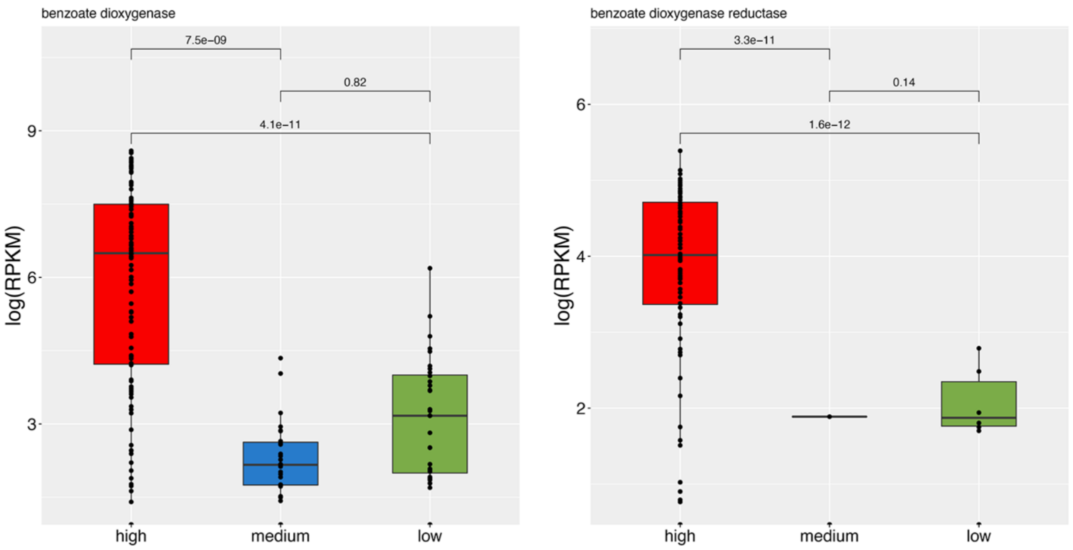

Figure S8. Box plots showing the abundance of genes related to benzoate degradation (log Reads Per Kilobase per Million, RPKM) in subjects from areas at HIGH, MEDIUM and LOW environmental pressure, as defined by MIEP index. The significance was tested by applying pairwise Wilcoxon test with p-value correction using the False Discovery Rate approach. Data are obtained from n= 82, 161 and 116 biologically independent samples from HIGH, MEDIUM and LOW environmental pollution groups, respectively.

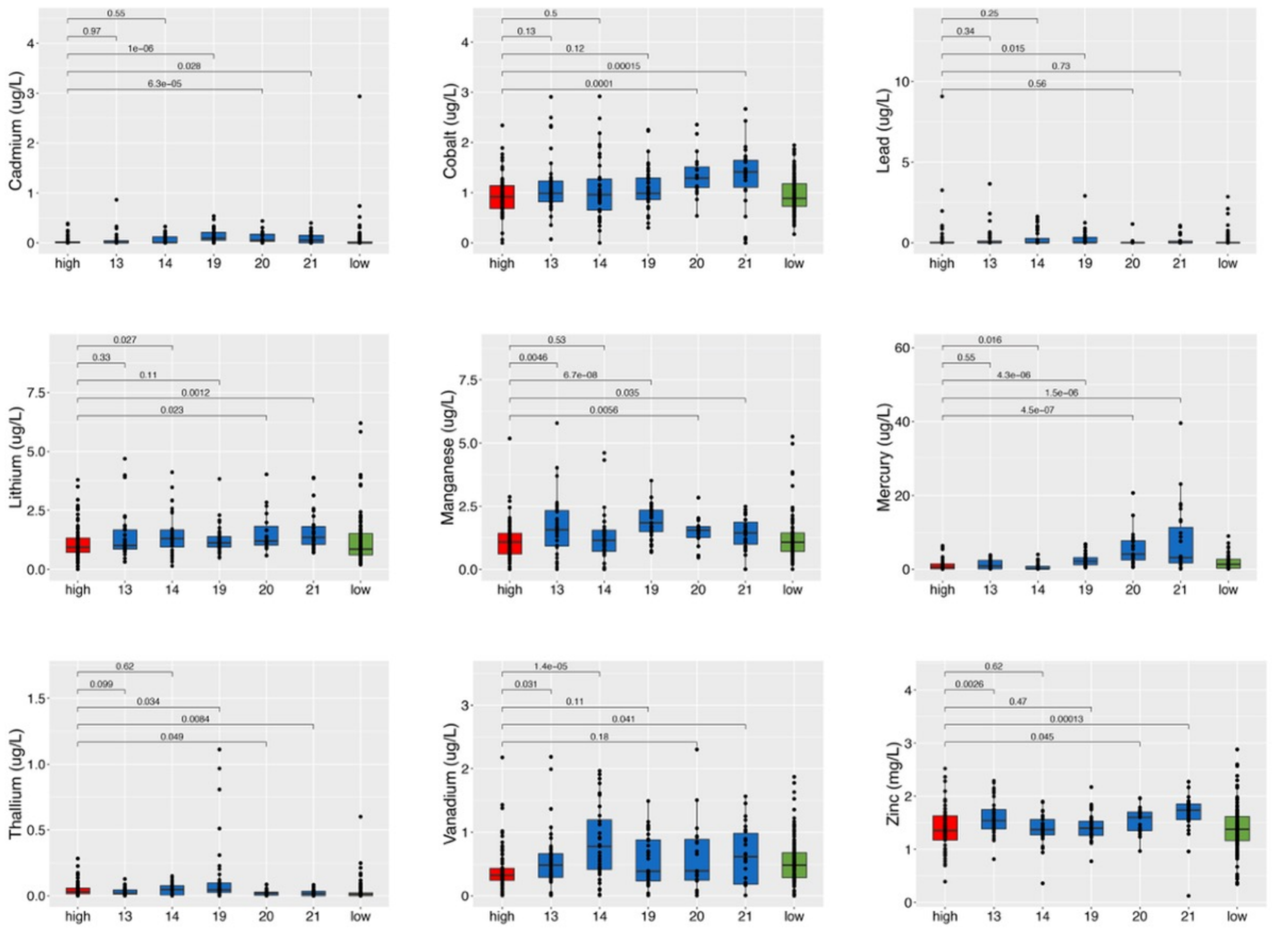

Figure S9. Box plots showing the plasmatic concentration of different heavy metals in subjects from areas at HIGH, MEDIUM and LOW environmental pressure, as defined by MIEP index. The MEDIUM group was further sub-divided according to the MIEP sub-clusters, as reported by Pizzolante et al., 2021. The significance was tested by applying pairwise Wilcoxon test with p-value correction using the False Discovery Rate approach. Data are obtained from n= 82, 161 and 116 biologically independent samples from HIGH, MEDIUM and LOW environmental pollution groups, respectively.

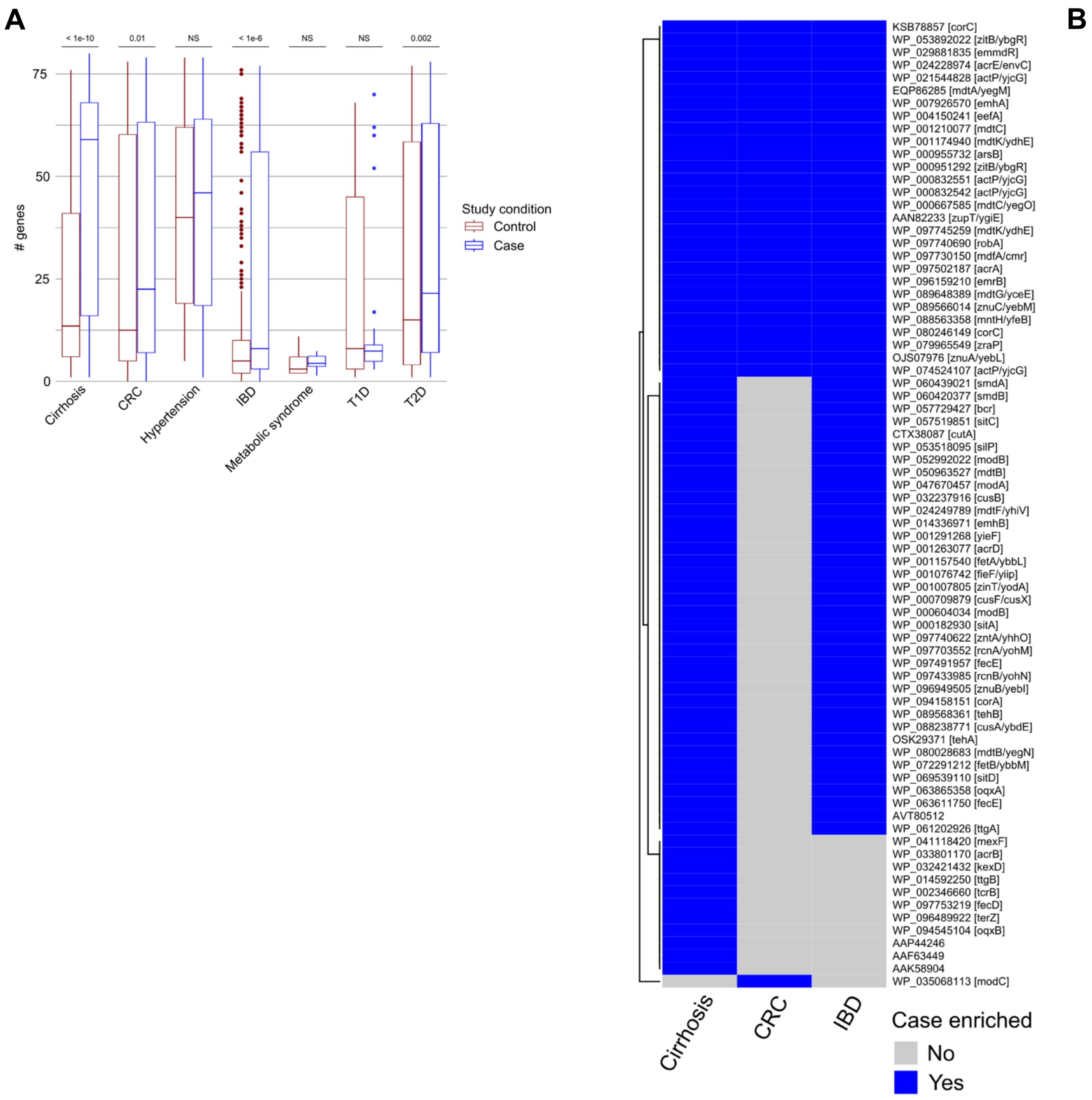

Figure S10. Box plots showing the number of genes from mibPOP and BacMet databases found in different case-control cohorts (A). Heatmap showing genes significantly enriched in cases/controls (B).

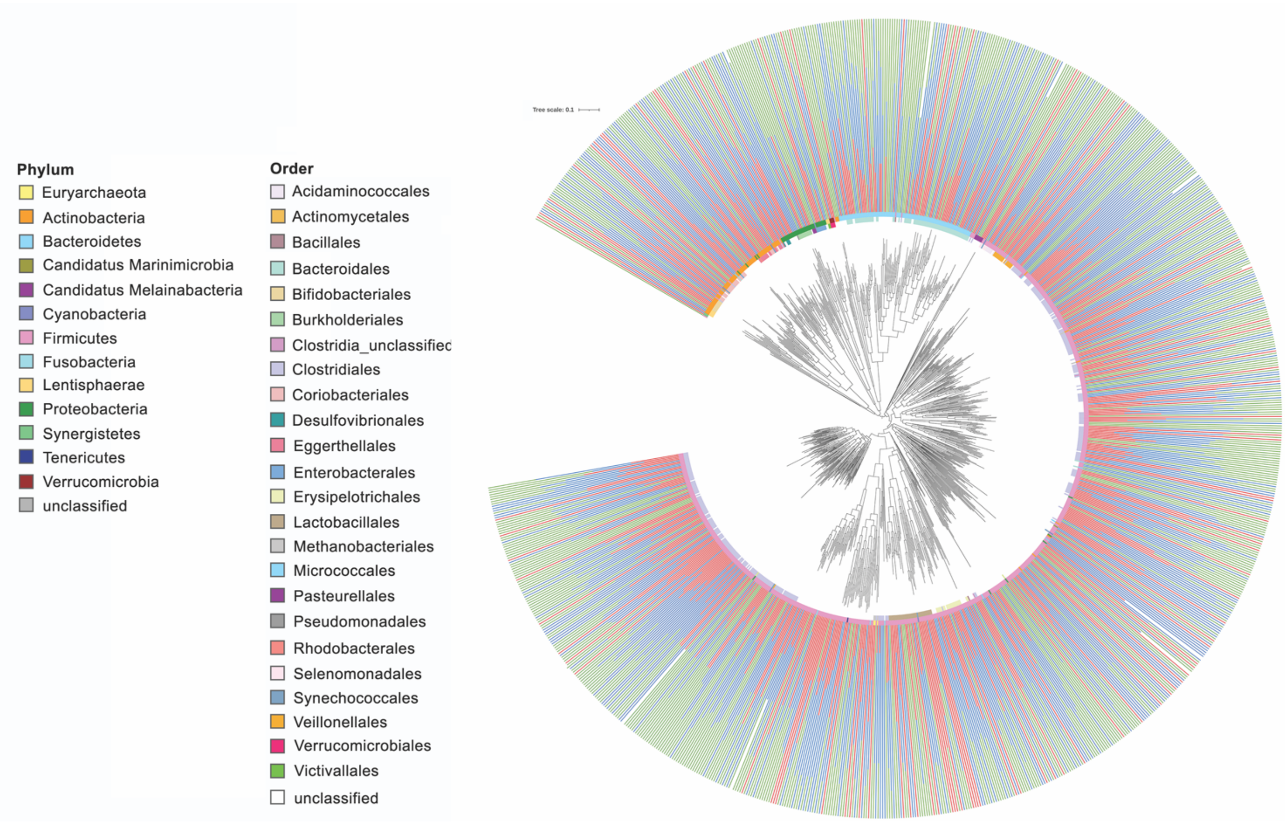

Figure S11. Phylogenetic tree of representative MAGs reconstructed in this study. Only one MAG for each SGB was randomly selected for the tree. Coloured rings indicate the phylum (P) or class (C) taxonomic classification. The bar chart indicates the proportion (%) of MAGs reconstructed from subjects belonging to HIGH (red), MEDIUM (blue) and LOW (green) environmental pressure groups.

SUPPLEMENTARY METHODS

Analysis of dioxins (PCDD/Fs) and polychlorinated biphenyls (PCBs)

The determination of PCDD/Fs and PCBs in human blood serum was carried out using a modified analytical method described by Brasseur et al. [1] The method allowed the determination of 17 PCDD/F and 12 DL-PCB congeners showing the highest toxicity for humans [2] and of 6 NDL-PCBs (PCBs 28, 52, 101, 138, 153 and 180). A volume of about 10-20 mL of serum was weighed, freeze-dried and extracted by an Accelerated Solvent Extraction (Dionex ASE 350, Thermo Fisher Scientific) system using a mixture of n-hexane/acetone as the solvent. The extract was cleaned up using an Extrelut NT3 column acidified with sulphuric acid 96% and eluted with a n-hexane/toluene mixture and subsequently with a florisil solid phase extraction (SPE) cartridge (Waters, Milford, MA, USA) eluted with dichloromethane.

For the HRGC-HRMS analysis of PCDD/Fs and PCBs, a DFS Magnetic Sector HRGC-HRMS system (Thermo Fisher Scientific, Waltham, MA USA) was used. A volume of 1 μL for both PCDD/Fs and PCBs was injected into the GCs in splitless mode, the temperature of the inlets was set at 280 °C.

The column used in the analysis of PCDD/Fs was a fused silica capillary column TR-1 (60 m × 0.25 mm i.d. × 0.1 μm, Thermo Fisher Scientific, Waltham, MA USA). The oven temperature was initially set at 100°C, which was maintained for 2 min; it was then increased to 220°C at a rate of 10°C min^{-1}, maintained for 10 min; and then increased at a rate of 5°C min^{-1} to 235°C and finally, after 7 min increased to 315°C at a rate of 18°C min^{-1}.

The column used in the analysis of PCBs was a fused silica capillary column HT8 (60 m × 0.25 mm i.d. × 0.25 μm, SGE Analytical Science, Victoria, Australia). The oven temperature program was 90°C for 1 min, rate 4 °C min^{-1} until 180°C, then rate 37.5°C min^{-1} until 285°C and finally increased to 320°C at 3°C min^{-1}.

The PCDD/F and PCB congeners were quantified using the isotope dilution method adding for each congener the corresponding $^{13}C_{12}$-isotope compound. For this purpose, before the extraction process,

each serum sample was spiked with a standard solution containing the $^{13}C_{12}$-labeled congeners. The data acquisition of the HRGC-HRMS analysis was performed using the multiple ion detection (MID) mode monitoring two isotopic masses for each PCDD/F and PCB congener to be quantified.

The concentrations of individual PCDD/F and DL-PCB congeners were expressed in pg g^{-1} on a lipid basis. The sums of 17 PCDD/F and 12 DL-PCB congeners were expressed in pg WHO-TEQ g^{-1} on lipid basis and were obtained from the toxic equivalent (TEQ) concentrations calculated using the toxic equivalent factors (TEFs) established for human risk assessment by the World Health Organization (WHO) [3].

The NDL-PCB congener concentrations and the sum of six NDL-PCBs were expressed in ng g^{-1} on lipid basis.

Trace elements analysis

The determination of metals and metalloids was carried out on the serum obtained from the volunteers. The analytical panel of trace elements includes arsenic (As), beryllium (Be), cadmium (Cd), cobalt (Co), chromium (Cr), copper (Cu), iron (Fe), mercury (Hg), lithium (Li), manganese (Mn), molybdenum (Mo), nickel (Ni), lead (Pb), antimony (Sb), selenium (Se), strontium (Sr), thallium (Tl), vanadium (V), and zinc (Zn). An inductively coupled plasma mass spectrometer ICP-MS NexION 350X (PerkinElmer, Waltham, USA) was used for determination of trace elements in human serum samples. The ICP-MS was equipped with a concentric nebulizer (Meinhard Associates, Golden, USA), a cyclonic spray chamber and a quartz torch with a quartz injector tube (2 mm i.d.).

The instrumental conditions were as follows: RF power of 1600 W, plasma gas flow rate of 18 mL min^{-1}, auxiliary gas (Ar) flow rate of 1.2 L min^{-1}, nebulizer gas flow rate of 1.0 L min^{-1}, plasma gas flow rate of 15 mL min^{-1}, a dwell time of 50 ms. Data were acquired in counts per second (cps). The following isotopes were selected: ^{75}As, ^{9}Be, ^{111}Cd, ^{59}Co, ^{52}Cr, ^{63}Cu, ^{57}Fe, ^{202}Hg, ^{7}Li, ^{55}Mn, ^{98}Mo, ^{60}Ni, ^{208}Pb, ^{121}Sb, ^{78}Se, ^{88}Sr, ^{205}Tl, ^{51}V and ^{66}Zn.

For each analyte, accuracy and precision were assessed by internal quality control and by using certified reference materials.

Superpure grade nitric acid 69% (v/v) was purchased from VWR International (Belgium). High purity deionised water (resistivity 18.2 MΩ cm) was produced in-house using a purification system Arium® pro (Sartorius, Germany). All glassware were soaked in a solution of nitric acid (10% w/v) then rinsed with high-purity water and dried prior to use.

For the analysis, 500 μL of serum was diluted 1:10 (v/v) with nitric acid 69 %. Calibration standard solutions and internal standards were prepared by successive dilution of a high purity ICP multi-element calibration standard solution of all 19 trace elements at 1000 mg L^{-1} obtained from Perkin Elmer (Norwalk, CT). A five-point calibration curve at suitable ranges (0.1-100 μg L^{-1}) was prepared daily in nitric acid 0.5% v/v for each element of interest and the internal standard was added on-line. The correlation coefficient (R^2) of calibration curves for all the trace elements was always greater than 0.99 showing a good linear relationship throughout the selected ranges of concentration. Each sample was analysed in duplicate, and the mean concentration was used in all statistical analyses.

The analytical method was validated by an in-house quality control procedure and appropriate quality assurance procedures and precautions were implemented in order to ensure the reliability of the results. Chemical blank determinations were analyzed for each very sample, to check for possible contamination. The limit of quantification (LOQ) for all the elements was calculated on the basis of the standard deviation of the intensity of twenty reagent blanks.

Metagenomics data analysis

Human reads were removed using the Human Sequence Removal pipeline developed within the Human Microbiome Project by using the Best Match Tagger (BMtagger; https:// hmpdacc.org/hmp/doc/HumanSequenceRemoval_SOP.pdf). Then, non-human reads were quality-filtered using PRINSEQ 0.20.4, trimming reads at the first occurrence of a base with a Phred score < 15. Reads shorter than 75 bp were discarded. Number of reads for each sample is reported in Supplementary Data 1. High-quality reads were imported in mOTUs2 [4] to obtain species-level, quantitative taxonomic profiles. The standard mOTUs database was used for taxonomic assignment. High-quality reads were assembled using MEGAHIT v. 1.2.2 [5] and contigs <1000 bp were

discarded. Genes were predicted from contigs by using MetaGeneMark v. 3.26 [6]. Assembly results are reported in Supplementary Data 5. We specifically focused on genes involved in the degradation pathways or in the resistance to dioxins and other environmental pollutants. Predicted genes were aligned (using DIAMOND v. 2.0.4 [7]) against genes coding for enzymes involved in persistent organic pollutants (POPs) biodegradation (as reported in the mibPOPdb [8], release 30.11.2021) and resistance to heavy metals (BacMet database v. 2.0 [9]). An e-value cutoff of $1e^{-5}$ was applied, and a hit was required to display >95% of identity over at least 50% of the query length. To obtain the gene abundance, short reads were mapped to the genes using Bowtie2 and the number of mapped reads was normalized using the RPKM method (reads per kilo-base per million mapped reads [10]). Microbial gene richness was estimated as described previously [11].

Contigs (>1000 bp) were also binned using MetaBAT2 [12] v. 2.12.1, and Metagenome Assembled Genomes (MAG) quality was estimated with CheckM [13] v. 1.1.3. Only MAGs with >50% completeness and <5% contamination were retained for further analyses. MAGs binned in this study were clustered to a genomic database including high-quality MAGs previously reconstructed from human metagenomes and NCBI RefSeq genomes using PhyloPhlAn3.0 [14]. Pairwise genetic distances between genomes were calculated using Mash (version 2.0; option "-s 10000" for sketching;). A Mash distance <5% from any of the database genomes was considered to place the MAG within the relative Species-level Genome Bin (SGB). When a MAG showed > 5% distance from any reference genomes, it was considered a novel species (unknown SGB, uSGB), and the taxonomic assignment was made at genus (> 5% and < 15% distance), family (> 15% and < 25% distance) or phylum (> 25% distance), using thresholds previously reported [15]. RAxML 8.0 was used to generate a phylogenetic tree, including one MAG for each SGB, which was visualized in iTOL [16] v. 5.5.1. A list of the MAGs reconstructed is reported in Supplementary Data 3.

The identification of BacMet and mibPOP in publicly available metagenomes was performed by considering 3,769 subjects from 24 datasets, collected in a previously published repository [17]. The datasets included case-control studies composed by healthy adult controls and subjects with different

diseases (liver cirrhosis, n=282 subjects; colorectal cancer, CRC, n= 1,395; hypertension, n=235; Inflammatory Bowel Disease, IBD, n=851; metabolic syndrome, n=15; type-1 and type-2 diabetes, n=173 and 818). Genes predicted from the metagenomic assemblies of these metagenomes were mapped against the BacMet and mibPOP databases as described before.

SUPPLEMENTARY REFERENCES

1. Brasseur C, Pirard C, Scholl G, *et al.* Levels of dechloranes and polybrominated diphenyl ethers (PBDEs) in human serum from France. *Environ Int* 2014;65:33-40. doi:10.1016/j.envint.2013.12.014
2. Van den Berg M, Birnbaum LS, Denison M, *et al.* The 2005 World Health Organization reevaluation of human and Mammalian toxic equivalency factors for dioxins and dioxin-like compounds. *Toxicol Sci* 2006;93(2):223-41. doi:10.1093/toxsci/kfl055
3. Esposito M, Serpe FP, Diletti G, *et al.* Serum levels of polychlorinated dibenzo-p-dioxins, polychlorinated dibenzofurans and polychlorinated biphenyls in a population living in the Naples area, southern Italy. *Chemosphere* 2014;94:62-69. doi:10.1016/j.chemosphere.2013.09.013
4. Milanese A, Mende DR, Paoli L, *et al.* Microbial abundance, activity and population genomic profiling with mOTUs2. *Nat Commun* 2019;10(1):1014. doi:10.1038/s41467-019-08844-4
5. Li D, Liu CM, Luo R, *et al*. MEGAHIT: an ultra-fast single-node solution for large and complex metagenomics assembly via succinct de Bruijn graph. *Bioinformatics* 2015;31(10):1674-6. doi:10.1093/bioinformatics/btv033
6. Zhu W, Lomsadze A, Borodovsky M. Ab initio gene identification in metagenomic sequences. *Nucleic Acids Res* 2010;38(12):e132. doi:10.1093/nar/gkq275
7. Buchfink B, Xie C, Huson DH. Fast and sensitive protein alignment using DIAMOND. *Nat Methods* 2015;12(1):59-60. doi:10.1038/nmeth.3176
8. Ngara TR, Zeng P, Zhang H. mibPOPdb: An online database for microbial biodegradation of persistent organic pollutants. *iMeta* 2022;1(4):e45. doi:10.1002/imt2.45
9. Pal C, Bengtsson-Palme J, Rensing C, *et al.* BacMet: antibacterial biocide and metal resistance genes database. *Nucleic Acids Res* 2014;42:D737-43. doi:10.1093/nar/gkt1252
10. Mortazavi A, Williams BA, McCue K, *et al*. Mapping and quantifying mammalian transcriptomes by RNA-Seq. *Nat Methods* 2008;5(7):621-8. doi:10.1038/nmeth.1226
11. Le Chatelier E, Nielsen T, Qin J, *et al.* Richness of human gut microbiome correlates with metabolic markers. *Nature* 2013;500(7464):541-6. doi:10.1038/nature12506

12. Kang DD, Li F, Kirton E, *et al.* MetaBAT 2: an adaptive binning algorithm for robust and efficient genome reconstruction from metagenome assemblies. *PeerJ* 2019;7:e7359. doi:10.7717/peerj.7359.eCollection2019
13. Parks DH, Imelfort M, Skennerton CT, *et al*. CheckM: assessing the quality of microbial genomes recovered from isolates, single cells, and metagenomes. *Genome Res* 2015;25(7):1043-55. doi:10.1101/gr.186072.114
14. Asnicar F, Thomas AM, Beghini F, *et al.* Precise phylogenetic analysis of microbial isolates and genomes from metagenomes using PhyloPhlAn 3.0. *Nat Commun* 2020;11(1):2500. doi:10.1038/s41467-020-16366-7
15. Pasolli E, Asnicar F, Manara S, *et al.* Extensive unexplored human microbiome diversity revealed by over 150,000 genomes from metagenomes spanning age, geography, and lifestyle. *Cell* 2019;176(3):649-62. doi:10.1016/j.cell.2019.01.001
16. Letunic I, Bork P. Interactive Tree Of Life (iTOL) v5: an online tool for phylogenetic tree display and annotation. *Nucleic Acids Res* 2021;49(W1):W293-6. doi:10.1093/nar/gkab301
17. Pasolli E, Schiffer L, Manghi P, *et al.* Accessible, curated metagenomic data through ExperimentHub. *Nat Methods* 2017;14(11):1023-4. doi:10.1038/nmeth.4468
