# Supplementary material for: Exposure to environmental pollutants selects for xenobiotic-degrading functions in the human gut microbiome
Source: Nat Commun. 2024 May 27;15:4482. doi: 10.1038/s41467-024-48739-7 (PMC11130323; doi:10.1038/s41467-024-48739-7)
Supplement: Supplementary file 3 — Description of Additional Supplementary Files [file 41467_2024_48739_MOESM3_ESM.pdf]

Description of Additional Supplementary Files

File name: Supplementary Data S1.
Description: Subject metadata and blood concentration of dioxins and heavy metals.

File name: Supplementary Data S2.
Description: List of significantly different taxa between HIGH, MEDIUM and LOW groups. p-value was computed using pairwise Wilcoxon's tests and corrected using the False Discovery Rate approach.

File name: Supplementary data S3.
Description: Number of MAGs reconstructed in this study and taxonomic identification.

File name: Supplementary Data S4.
Description: Number of reads and assembly statistics
